# Targeting cancer with peptide aptamers

**DOI:** 10.18632/oncotarget.297

**Published:** 2011-06-24

**Authors:** Renaud Seigneuric, Jessica Gobbo, Pierre Colas, Carmen Garrido

**Affiliations:** ^1^Heat Shock Proteins and Cancer, INSERM, UMR 866 IFR 100, Faculty of Medicine, 7 Boulevard Jeanne D'Arc, 21000 Dijon, France; ^2^Université de Bourgogne, Dijon, France; ^3^CNRS USR 3151, P2I2 Group, Station Biologique, Roscoff, Bretagne, France

**Keywords:** Cancer, targeted therapy, peptide, aptamer, heat shock protein

## Abstract

A major endeavour in cancer chemotherapy is to develop agents that specifically target a biomolecule of interest. There are two main classes of targeting agents: small molecules and biologics. Among biologics (e.g.: antibodies), DNA, RNA but also peptide aptamers are relatively recent agents. Peptide aptamers are seldom described but represent attractive agents that can inhibit a growing panel of oncotargets including Heat Shock Proteins. Potential pitfalls and coming challenges towards successful clinical trials are presented such as optimizing the delivery of peptide aptamers thanks to Nanotechnology.

Rational drug design is the quest, launched by Paul Ehrlich about a century ago, for designing a ‘magic bullet’: a targeting agent that specifically interacts with, and mostly inhibits, a biomolecule of interest. Essentially, this biomolecule is an intracellular or membrane-bound protein identified as contributing to a disease state. The choice of the protein to be targeted is constrained by our ability obtain highly specific, bioactive ligands. Usually, for small molecules, a requirement is the presence of a pocket (e.g.: the ATP-binding cavity of protein kinases) where the targeting agent can fit snugly and establish the multiple low-energy interactions in 3 dimensions that confers its (high) affinity and specificity.

Within the last 10 years[1], a number of such targeting agents have been discovered by screening or rational design. They fall in 2 classes: small molecules and biologics. The former are developed by (pharmaceutical) chemists, with imatinib as an archetype. The latter originate from molecular biologists and are typically represented by monoclonal antibodies (mAbs) such as trastuzumab or cetuximab. Due to their size (molecular weight <500Da[2]), small molecules are relatively easy to synthesize, can diffuse well (including through the membrane bilayer) and are generally orally available[3]. On the other hand, mAbs are large (~150kDa[4, 5]) and complex molecules[6] that are delivered intra-tumorally or intravenously[3]. To reduce the immune response they inherently trigger, efforts were devoted to design new generations of antibodies switching from murine antibodies to: chimeric (~60% human, example: Cetuximab[7]), humanized (~90% human, e.g.: Trastuzumab) or human antibodies (100%, e.g.: Panitumumab). Up to now, biologics have in fact a higher rate of approval success rate (18% for chimeric and 24% humanized monoclonal antibodies) than new chemical entities including small molecules (5%)[3, 8] especially in oncology[3].

## A NEW TOOL: PEPTIDE APTAMERS

Among biologics, aptamers (a word combining the Latin *aptus* ‘fitting’ with the Greek *meros* ‘part’[9]) are emerging as a new class of targeting agents. Aptamers (i.e.: DNA, RNA aptamers) but also peptide aptamers are indeed apt to specifically inhibit biomolecules of interest with high affinity[9-11].

**Figure 1:**
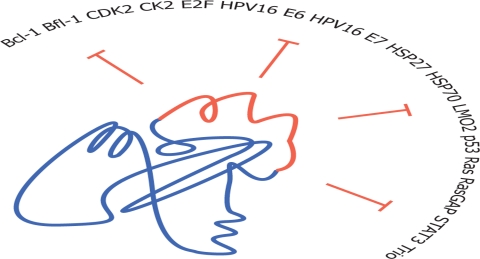
Oncotargets of peptide aptamers The typical structure of a peptide aptamer is composed of a constant scaffold (in blue, e. g. : the bacterial protein thioredoxin A) with an inserted short variable random region (in red). A panel of oncotargets inhibited by peptide aptamers is provided.

Peptide aptamers consist of a short (~10-20 amino acids), conformationally constrained[12] variable random peptide sequence inserted into a scaffold protein (most often the bacterial protein thioredoxin A[12, 13]) as shown in figure 1. They can thus be considered as miniaturized, simplified antibodies[12, 13]. A unique feature of peptide aptamers relies in their doubly constrained target-binding loop[13], compared to other man-made biomolecules that consist of peptidic sequences fused terminally to a carrier protein. Constrained conformations[13-15] require less energy to bind the target of interest, resulting in peptide aptamers with high affinities and *K*^D^ values in the 10 – 100 nM range[10, 12]. Compared to monoclonal antibodies, DNA, RNA and peptide aptamers are relatively small, weighting ~10-20kDa[16] with reduced immunogenicity compared to antibodies[9].

## TRANSLATION TO THE CLINICS: OF MICE AND MEN

Of the ~108,400 recorded clinical trials (http://clinicaltrials.gov/, last access: the 8^th^ of June 2011), ~5,500 are involving targeted therapies, with ~1,700 in the field of oncology. Among these, there are ~500 trials assessing peptides but only 3 studies concerning aptamers (none with peptide aptamers). There are at least 2 interesting approaches to be enlightened. One is the success of Pegaptanib sodium (Macugen, Eyetech Pharmaceuticals/Pfizer), the first FDA-approved aptamer (December 2004) for use in humans. As with other types of agents, it took about a decade of preclinical development to improve and characterize its biological effects[17]. Nevertheless, this anti-VEGF pegylated RNA aptamer developed for the treatment of neovascular age-related macular degeneration is now involved in ~50 trials. Another interesting example includes AS1411 (4 clinical trials) for the study of Acute Myeloid Leukemia (2 clinical trials), Renal Cell Carcinoma (1 clinical trial) and advanced tumors (1 clinical trial). This DNA aptamer targeting nucleolin induces apoptosis and may be combined with cytarabine to obtain a synergistic effect. It is the first-in-class DNA aptamer anticancer agent, and is currently in phase II clinical trials including solid tumours[18].

More recently developed, peptide aptamers are to date tested in preclinical models only. In the context of oncology, *in vitro* and/or *in vivo* data indicate that peptide aptamers are able to inhibit a growing panel of oncotargets including: the human papillomavirus HPV16 E6 oncoprotein[19] in 2000, ErbB2 receptor[20], mutant p53[21], but also HSP70[22] and HSP27[23] within these last months. So what are the foreseen barriers towards successful clinical trials?

### Reaching the tumor

To date, peptide aptamers target the cancer cells via different means. The two most frequent delivery options are liposome-like chariots and protein transduction domains (PTDs). Potential issues of the former approach might be related to toxicity and stability. Concerning the later, the most widely used PTD, derived from HIV-TAT protein, is a positively charged sequence that represents an interesting approach. However, it might affect protein structure. Far from trivial, delivering the targeting agent specifically to the desired site of action will more than probably benefit from nanotechnology. Several ongoing clinical options exist. They include drug delivery systems composed of: lipid nanoparticles, albumin-based, micelles, polymer-based or gold nanoparticles[9] that should be investigated for peptide aptamers.

### Dose calculations

Different from organic small molecules by nature, dose calculations for biologics nevertheless remain almost exclusively traditional[24] even though they exhibit non-linear dose-response curves[6]. As targeting agents are often combined with cytotoxics (e.g.: doxorubicin, cisplatin or taxol), drug combinations need careful evaluation. Also, the design of clinical trials for targeting agents should be revisited accordingly[25].

### Early detection

Obviously, the earlier the disease is detected, the greater the chances of curing it. Efforts should be devoted to improve current detection limits. Also, it is necessary to develop and validate appropriate biomarkers for cancer diagnosis as well as treatment follow-up[26]. Interestingly, aptamers can be functionalized onto sensing schemes for biodetection[9] with the emerging ‘aptasensors’[27].

### Patient selection: when one treatment does not fit all

Related to early detection is the identification of the right panel of patients. Cancer is known to be a highly heterogeneous disease. For instance, the EGFR pathway is disrupted in fewer than 15% of patients with lung cancer[28]. One of the 4 EGFR family member, HER2, is overexpressed in ~30% of breast cancers[6, 29]. Thus, averaging the response of a few responders in an overall non-responding group would be cancelled out and the development of companion diagnostics to eventually identify suitable patients is mandatory.

### Side effects: evolution for every one

Since “*Nothing makes sense in medicine except in the light of biology*”[30] and also that “*Nothing in biology makes sense except in the light of evolution*”[30], it may prove useful to get insights from an evolutionary process that shaped us over a couple of billions of years. The quest for new agents have led to some disastrous clinical trials like CAST and SWORD in cardiology[31]. Some of the lessons learned are that searching for druggable portions of different types of ion channels (e.g.: sodium, potassium) may lead to a common –well conserved- protein domain (e.g.: the pore-forming modules[32]) that is shared by those different types of channels[33], potentially leading to undesired effects and complex cardiac patterns[31], sometimes lethal. In cancer, a similar pitfall was recently noticed when assessing the selectivity of erlotinib hydrochloride, gefitinib and imatinib for instance[29]. Targeting ATP-pockets is actually a very common trend in rational drug design since these pockets exhibit high druggability indexes and may be seen as a bias from the concept of druggability. Therefore, when intending to design a specific inhibitor, one should also evaluate sets of related targets which contain similar pockets. For protein kinases for instance, the human kinome space was found to consist of 518 kinases[34]. As a general guideline, a threshold of 60% sequence identity was determined for the ATP-pocket that differentiated between binding sites of related kinases versus variations in the binding sites of the same kinase[35]. This indicates that kinases with a sequence identity >60% have a high probability of being inhibited by the same compounds[35]. Peptide aptamers were in fact developed to circumvent the requirement of targeting pockets and to address other types of target sites, non-druggable by small molecules. They can inhibit protein function via the disruption of protein-protein interactions or a number of other mechanisms[36]. In the context of protein kinases, inhibitory peptide aptamers would be expected to be more specific than their small molecules inhibitors, thereby reducing side-effects related to off-targets inhibition.

## CONCLUDING REMARKS

The attrition rate in clinical trials remains high, especially in cancer[8]. One major reason probably relies in the intrinsic heterogeneity of tumors that harbour several mutations[37, 38]. Therefore, instead of heading for the design of the magic bullet, one may in fact want to broaden the spectrum of targets, and move towards polypharmacology as interaction networks play an increasing role in our current understanding of drug efficacy and side effects[39-41]. It was indeed found for instance that one of the archetypes of targeted therapy, imatinib, inhibits other proteins as well and is thus in fact not perfectly specific[39]. Alternatives to ‘dirty’ or promiscuous drugs intended to target and inhibit a set of pathways disrupted in cancer include targeting a molecular chaperone, ideally an inducible heat shock protein[22, 23, 42]. The rational is that cancer cells, because they must extensively rewire their metabolic and signalling networks, need for their survival an abundant content of chaperones like the inducible HSP70 or HSP27 that are no or hardly expressed in normal cells. This general cancer cells’ addiction to heat shock proteins make the recently described HSP70 and HSP27 peptide aptamers all the more interesting as sensitizing agents in cancer therapy. However, the use of peptide aptamers as targeting agents in cancer therapy need to be carefully validated since the existing data is only preclinical.
